# Medical Department of Niagara University

**Published:** 1890-10

**Authors:** 


### MEDICAL DEPARTMENT OF NIAGARA UNIVERSITY.

The Medical Department of Niagara University began its eighth annual course of lectures on the 22d of September, ult. The opening exercises were held in the evening in the new amphitheatre and were largely attended by new and old students, alumni, and friends of the school. The address of the evening was delivered by Dr. Floyd S. Crego, Professor of Nervous Diseases and Insanity.

After extending a hearty welcome to all, the doctor reviewed the success of the school, and spoke in detail of its aims and methods ; he also called especial attention to the new law creating a State Board of Medical Examiners, which he said would receive the hearty support of the school, as it had always advocated whatever advanced the standard of medical education.

The larger part of the address was devoted to the consideration of the advances that had recently been made in medicine, especially in the line of chemistry, pathology, and diseases of the nervous system.

The regular order of lectures opened on the 23d, with a good class in attendance.

The additions to the college which have been in progress during the Summer are nearly finished, and the building, as a whole, is one of the most complete of its kind in the country. As now enlarged, it provides a spacious and beautiful amphitheatre, two lecture rooms, pathological, physiological, and chemical laboratories, dissection room, waiting-room, museum, library, dispensary apartments, and several smaller and private rooms. The faculty, alumni, and their friends feel a just pride in these improvements and increased accommodations, and these in turn reflect much credit upon the zeal and earnestness of those who have projected and maintained this institution and the principles for which it stands.

---

